# Validation of an algorithm based on clinical, histopathological and immunohistochemical data for the diagnosis of early-stage mycosis fungoides^☆^^☆☆^

**DOI:** 10.1016/j.abd.2020.01.002

**Published:** 2020-03-20

**Authors:** Gustavo Moreira Amorim, Daniele Carvalho Quintella, João Paulo Niemeyer-Corbellini, Luiz Claudio Ferreira, Marcia Ramos-e-Silva, Tullia Cuzzi

**Affiliations:** a Postgraduate Program in Anatomical Pathology, Faculdade de Medicina, Universidade Federal do Rio de Janeiro, Rio de Janeiro, RJ, Brazil; b Department of Pathology, Faculdade de Medicina, Universidade Federal do Rio de Janeiro, Rio de Janeiro, RJ, Brazil; c Dermatology Service, Hospital Universitário, Universidade Federal do Rio de Janeiro, Rio de Janeiro, RJ, Brazil; d Instituto Nacional de Infectologia, Fundação Oswaldo Cruz, Rio de Janeiro, RJ, Brazil; e Discipline of Dermatology, Departamento de Clínica Médica, Faculdade de Medicina, Universidade Federal do Rio de Janeiro, Rio de Janeiro, RJ, Brazil

**Keywords:** Diagnosis, Immunohistochemistry, Mycosis fungoides, Pathology

## Abstract

**Background:**

Diagnosis of mycosis fungoides is challenging due to the non-specificity of clinical and histopathological findings. The literature indicates an average delay of 4–6 years for a conclusive diagnosis. Refinement of the histopathological criteria for the diagnosis of patients in early stages of the disease is considered of interest.

**Objectives:**

To study the histopathological aspects of early-stage mycosis fungoides and the applicability, in a retrospective form, of the diagnostic algorithm proposed by Pimpinelli et al.

**Methods:**

Observational, retrospective, transversal study based on revision of histopathological exams of patients with suspected mycosis fungoides. Medical records were reviewed, and complementary immunohistochemistry performed.

**Results:**

Sixty-seven patients were included. The most frequent histopathological features were superficial perivascular lymphoid infiltrate (71.6%), epidermotropism (68.7%), lymphocytic atypia (63.8%), hyperkeratosis (62.7%) and acanthosis (62.7%). Forty-three patients scored 4 points at the algorithm, by clinical and histological evaluation. Immunohistochemistry was performed on 23 of the 24 patients with less than 4 points. Of those 23, 22 scored 1 point, allowing a total of 61 patients (91%) with the diagnosis of early-stage mycosis fungoides.

**Study limitations:**

Its retrospective character, reduced sample size and incomplete application of the algorithm.

**Conclusions:**

Application of the Pimpinelli et al. algorithm, even in an incomplete form, increased the percentage of cases diagnosed as mycosis fungoides. Routine application of the algorithm may contribute to earlier and specific management and improvement of the patients’ outcome.

## Introduction

Diagnosis of mycosis fungoides (MF) is challenging.^1^ The literature indicates an average delay of 4–6 years for it to be stablished,^2^, ^3^, ^4^ and clinico–pathological correlation is critical.^5^ Pimpinelli et al. proposed an algorithm for early stage MF diagnosis^6^ that provides a score based on clinical, histopathological and immunohistochemical findings, as well as investigation of the clonal T-cell receptors (TCR) gene rearrangement. Application of the algorithm was endorsed by the International Society for Cutaneous Lymphoma (ISCL) and was considered in recently published consulted review articles.^1^, ^5^

Aiming to study the histopathological aspects of early stages of MF and to confirm the applicability of the algorithm proposed by Pimpinelli et al., we reviewed the first histopathological exams performed on patients with early-stage MF who had been followed-up and treated in the Photodermatology Outpatient Clinic at the University Hospital of the Federal University of Rio de Janeiro.

## Methods

This was an observational transversal study, based on the review of first histopathological exams performed on adult patients (18 years old or older) with MF, diagnosed, treated and followed up (5 years minimum) in the Photodermatology Outpatient Clinic at the University Hospital, from January 2000 to December 2015.

All patients included in the study had a final diagnosis of MF, established during their follow-up period by the following criteria:

- typical clinical evolution (progression of macular lesions to plaques and even tumors in some cases), according to the so-called classical Alibert-Bazin form;^5^, ^7^

- typical findings in subsequent histopathological exams (epidermotropism of atypical lymphocytes and Pautrier's microabscesses).^8^

Patients were classified as being in early-stage according to the ISCL and the European Organization for Research and Treatment of Cancer (EORTC) propositions. They correspond to disease in stage IA (T1N0M0), IB (T2N0M0) or IIA (T1 or 2N1 or 2M0) according to TNMB.^9^

Only cases with available paraffin blocks for immunohistochemical analysis were included.

Two experienced dermatopathologists, who had no knowledge of the original report, reviewed at same time each patient's initial histopathological exam, and accorded the analysis of the specific established histological parameters. All samples corresponded to 4–5 mm punch biopsies. Patients with more than one sample were evaluated considering the joint analysis of the samples to conclude for a compatible MF diagnosis or not. Only hematoxylin–eosin stained sections were evaluated, and paraffin blocks were selected for subsequent immunohistochemical study.

Clinical data was collected with the objective of characterize the study population, and explore the clinical criteria proposed by Pimpinelli et al.^6^ Staging was analyzed in a qualitative ordinal mode, according to the prevailing TNMB staging protocol.^9^

Immunohistochemistry (Pathology Service, Evandro Chagas National Institute of Infectology/FIOCRUZ) was applied when clinical and histopathological criteria did not score the 4 points necessary for the diagnosis.^6^ Tissues sections reacted with CD3 (rabbit polyclonal, 1/100, Cell Marque™), CD2 (MRQ-11, mouse monoclonal, 1/50, Cell Marque™), CD5 (SP19, rabbit monoclonal, 1/100, Cell Marque™) and CD7 (EP132, mouse monoclonal, 1/100, Cell Marque™) as primary antibodies. To characterize epidermal disagreement, only the samples with CD3^+^ epidermal lymphocytes were considered, in order to ensure presence of the cells in that location. Investigation of clonal T-cell receptors (TCR) gene rearrangement is not available in our laboratory at this time.

The considered histopathological, clinical and immunohistochemical variables are presented in a table as supplemental on line content. The data were analyzed with Excel 2011 (Microsoft® Excel® for Mac 2011/Version: 14.2.0). The analysis was descriptive.

The study followed the Resolution 466/12 of the Brazilian National Health Council, was registered in Plataforma Brasil and was approved by Committee of Ethics in Research of the HUCFF/UFRJ (CAAE 59235916.9.0000.5257).

## Results

From an initial number of 102 patients with early diagnosed MF, 67 had histopathological exams with paraffin blocks available for immunohistochemical analysis. All 67 were included since follow-up confirmed a MF diagnosis according to study inclusion criteria.

Men and women were almost equally affected. The mean age founded was 53.27 years. The majority of patients presented with multiple lesions of variable sizes (98.5%), affecting photo-protected areas (98.5%), with chronic and progressive evolution (the mean time between the onset of symptoms and the final diagnosis was 51.31 months, ranging from 2 to 360 months). Most patients had plaque lesions (64.2%). Poikiloderma was found only in 14.8% of the cases. Stage IB represented 52.2% of the sample. Mean time of follow up of 8.25 years.

**Figure 1:**
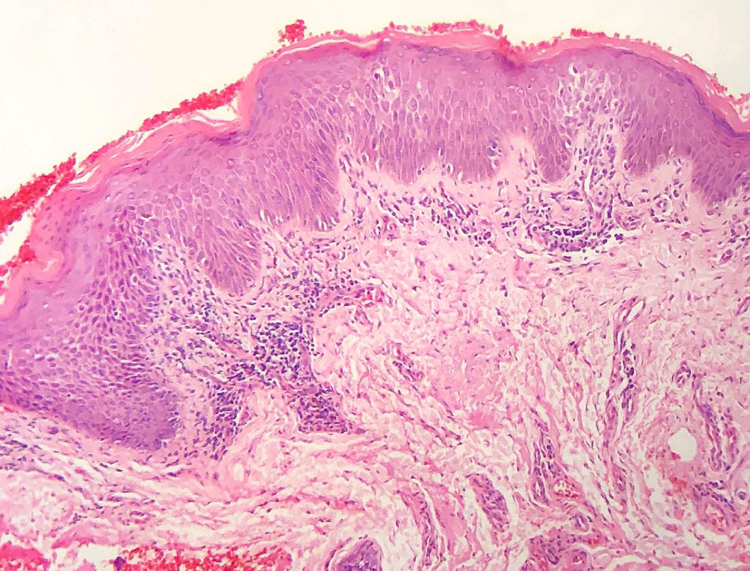
Superficial perivascular lymphoid infiltrate and insufficient amount of lymphocytes in the epidermis to characterize epidermotropism. In addition, there are hyperkeratosis, parakeratosis and acanthosis (Hematoxylin & eosin, x100).

**Figure 2:**
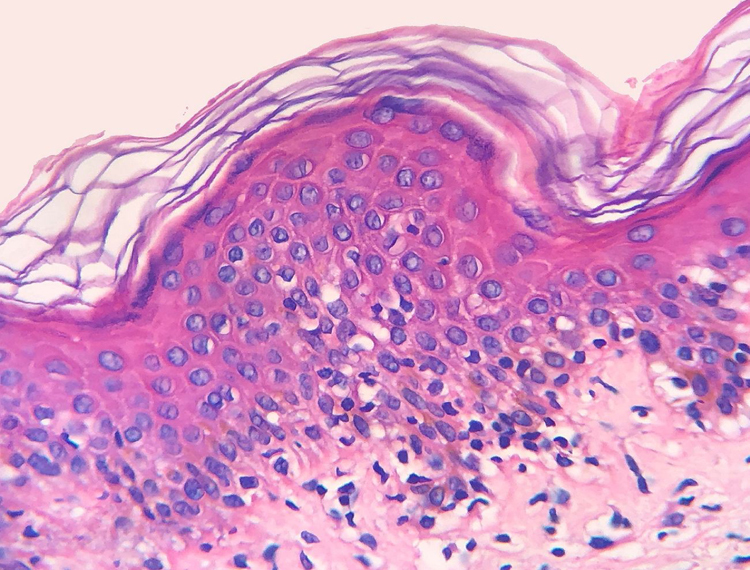
Atypical lymphocytes, with increased nuclear size, located along dermoepidermal junction and in the suprabasal cell layers. Some are haloed or present cerebriform contour (Hematoxylin & eosin, x400).

The frequency of the histopathological variables for assessment of biopsies with suspicion of MF, are presented in table 1. Superficial perivascular lymphoid infiltrate, epidermotropism without Pautrier's microabscesses, lymphocytic atypia, specially expressed by the increase of nuclear size of lymphocytes located in the epidermis, hyperkeratosis and acanthosis were the predominant histopathological findings (Figure 1, Figure 2).

**Table 1:** Histopathological characterization of the sample.

*Corneal layer alterations*		
Hyperkeratosis	62.7%	42/67
Parakeratoses	38.3%	23/67

*Epidermis*		
Normal thickness	25.4%	17/67
Thinned	3.0%	02/67
Irregular acanthosis	62.7%	42/67
Psoriasiform acanthosis	9.0%	06/67
Vacuolar alteration of the basal layer	9.0%	06/67

*Lymphoid infiltrate*		
Perivascular superficial	71.6%	48/67
Perivascular superficial and deep	6.0%	09/67
Lichenoid	14.9%	10/67
Diffuse and confluent^a^	4.5%	03/67
Compromising the hypodermis^b^	1.5%	01/67

*Epidermotropism*	68.7%	46/67
*Pautrier's microabscesses*	11.9%	08/67
*Folliculotropism with mucinosis*	4.5%	03/67
*Folliculotropism without mucinosis*	3.0%	02/67
*Pigmentary incontinence*	58.2%	39/67
*Lymphocytic atypia*	63.8%	44/67
*Convolution of epidermal nuclei*	20.3%	14/67
*Convolution of dermal nuclei*	0	0/67
*Increase of nuclear size in the epidermis*	55.1%	38/67
*Increase of nuclear size in the dermis*	18.8%	13/67
*Multinucleated giant cells*	3.0%	02/67
*Eosinophils*	7.5%	05/67
*Papillary dermal fibroplasia*	74.6%	50/67

a In 3 cases, the diffuse and confluent pattern was focal, so that the perivascular superficial and deep pattern prevailed.

b In the only case where the infiltrate extended focally to the hypodermis, the prevailing pattern was perivascular superficial and deep.

**Figure 3:**
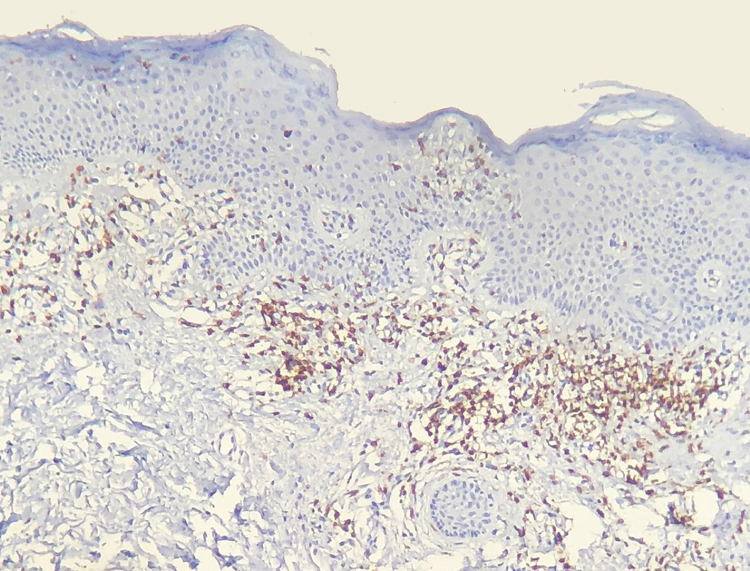
CD3^+^ cells comprise most of dermal infiltrate and are also seen in the epidermis (Immunohistochemistry, x40).

**Figure 4:**
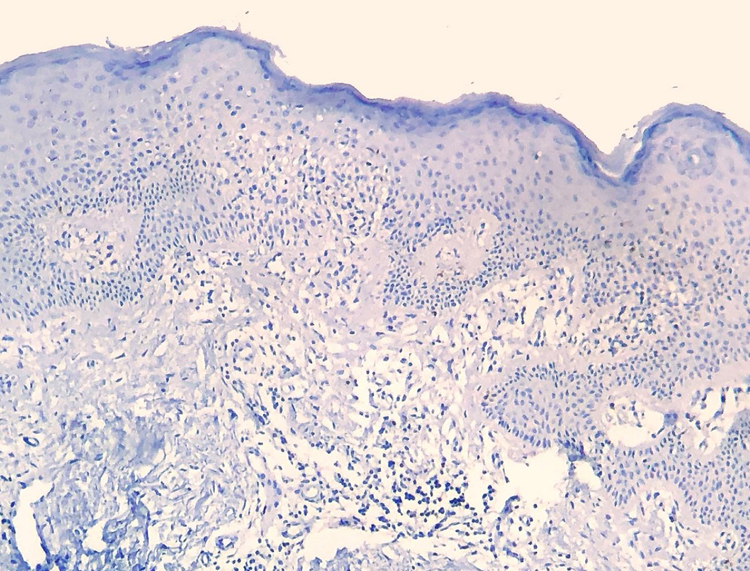
CD7 reaction is negative in both compartments, characterizing loss of that T-cell marker and dermoepidermal disagreement, regarding CD3 positivity (Immunohistochemistry, x40).

Results of immunohistochemical study performed on 23 out of 24 eligible samples are shown in tables as supplemental on line content. One of the selected paraffin blocks did not provide enough tissue to complete reactions. Decrease in the expression of T-cell markers and dermoepidermal disagreement (Figure 3, Figure 4) were observed in most of the cases.

The diagnostic impression based on review of histopathology together with the data regarding the modified application of the Pimpinelli et al. algorithm is shown in table 2.

**Table 2:** Comparative diagnostic rate between the histopathological review and the criteria of the algorithm adapted from Pimpinelli et al.

Patients	Criteria	(%)	*n*
Compatible with MF diagnosis	Histopathological review	64.2%	43/67
4 points at algorithm	Clinical + Histopathological	64.2%	43/67
	Clinical + Histopathological + Immunohistochemical	91.0%	61/67
<4 points at algorithm	Would require investigation of clonal rearrangement of the TCR	9.0%	06/67

## Discussion

An observational study of transversal design was performed, based on review of histopathological exams from patients with a diagnostic suspicion of MF, who during the follow-up period had MF diagnosis undoubtedly confirmed. Histopathological exams were complemented with immunohistochemical profile with the objective to apply the diagnostic algorithm proposed by Pimpinelli et al. endorsed by recent review articles,^1^, ^5^, ^6^ without TCR clonality test.

From the clinical standpoint, MF presentation followed what is described in the literature, with more commonly multiple macules/patches and/or plaques of variable sizes, affecting photo-protected areas with chronic and progressive evolution (average evolution period: 51.31 months).^1^, ^5^, ^6^, ^7^, ^10^ This profile is in accordance with what is proposed by Pimpinelli et al. in their identification algorithm for early-stage MF; so that only one patient (1.5%) scored 1 point, while the remainder fulfilled the maximum 2 points. This demonstrates the good correlation between what we clinically identified with what is proposed by the algorithm.^6^

The histopathological review identified alterations of the corneum layer, with hyperkeratosis and parakeratosis which are common alterations in the initial MF stages, especially when taking into account the complaint of pruritus and the presence scaling on patch and plaque lesions.^2^, ^7^, ^8^

The predominant tissue reaction pattern was that of a superficial perivascular lymphoid infiltrate with pigmentary incontinence and papillary dermal fibroplasia, while lichenoid lymphoid infiltrate together with the superficial and deep perivascular pattern, and the diffuse and confluent pattern were uncommon. Higher percentages of lichenoid infiltrate were described in the studies by Nagaraghi et al. and Massone et al.^11^, ^12^ However, our findings support that only patients with initial MF stages, with low tumor load, were included.^2^, ^13^

Epidermotropism was present in 68.7% of analyzed patients, indicated by the alignment of haloed lymphocytes at the dermoepidermal junction or in a suprabasal location, without spongiosis. The alignment of lymphocytes along the basal keratinocytes is a finding in the histopathological evaluation of MF exams in early-stage^14^ and was described by Sanchez and Ackermann, in 1979, as criterion for its diagnosis.^11^, ^15^ In contrast, folliculotropism and Pautrier's microabscesses were rare, as proposed in literature.^2^, ^13^, ^14^ However, a large study conducted by Massone et al., with evaluation of 427 patients with MF in early stage, demonstrated higher percentages of Pautrier's microabscesses (19%), similar to the findings of Nagaraghi et al. (present in up to 66% of patients with plaque as clinical elementary lesion).^11^, ^12^

Lymphocytic atypia, based on the increased size and/or cerebriform contour of nuclei, was identified in 63.8% of patients. Valorization of cytological criteria, such as hyperconvoluted nucleus or increase in their size, for cells located in the epidermis or dermis is disputed in the literature and seems to be considered in more recent publications.^16^, ^17^, ^18^ Our findings showed that lymphocytic atypia was almost as frequent as epidermotropism, being in accordance with the analysis of recently mentioned authors. On the other hand, Massone et al. identified atypical lymphocytes only in 9% of patients, leading them to propose that architectural criteria, involving the infiltrate distribution pattern associated to the finding of epidermotropism, would be more relevant in the initial MF stage.^7^

The histopathological review considered the findings compatible with an MF diagnosis in 64.2% of cases. Thus, in 35.8% (24 of 67) of the cases, the histopathological exam, by itself, did not allow to diagnose MF. In fact, false negative rates in a first exam reach 40%.^19^ However, besides an unspecific histopathological picture, it is important to emphasize the lack of findings to establish another specific diagnosis, such as eczemas or psoriasis. So, in 24 patients in whom a diagnosis of MF could not be done, it could not also be excluded.

Still regarding these 24 cases, the main aspect challenging the MF diagnosis was the scarcity of infiltrate, a finding compatible with early disease, in concordance with non-infiltrated clinical lesions.^2^, ^13^, ^16^ Furthermore, histopathology findings could me masked by the use of medications, such as topic corticosteroids, that minimize the lymphocytic infiltrate and reduce lymphocytes from the dermo-epidermal junction. Considering that these medications are easily accessible, bought without prescription, we frequently observe in our clinical practice that patients make inadvertent use of that group of substances. Patients with clinical suspicion of MF should ideally stop using topic steroids as well as systemic immunosuppressants (if it is the case) 2–4 weeks before carrying out a biopsy for not impairing the histopathological analysis, while emollients can be continued.^6^ The retrospective design of the study makes it difficult to determine if there was use of medications at the time of the first biopsy, representing therefore a limitation to be considered.

The decrease in the expression of T-cell markers, such as CD2, CD3, CD5 and CD7, in MF patients is based on the idea that as the disease progresses, a predominant abnormal phenotype is identified.^6^, ^13^ Reduction of CD2, CD3 and/or CD5in at least 50% of lymphoid cells is an importantly sensitive criterion for identification of T-cell lymphomas.^20^ Loss of positivity seems to occur initially in epidermal lymphocytes, and later in those located in the dermis.^6^, ^20^ Among CD2, CD3, CD5 and CD7, apparently the most specific criterion would be the reduction of CD7 positivity to less than 10% of the lymphoid cells.^6^, ^21^

Applying the immunophenotypic analysis on 23 cases that did not meet criteria for diagnosis, 22 scored in the algorithm. The most prevalent criterion was the reduction of positivity to less than 50% of the infiltrate for CD2 and/or CD5, being therefore, in this analysis, the most sensitive criterion, similar to what is proposed in literature.^6^, ^20^ Finally, 13 cases presented the supposedly more specific criterion, with reduction of CD7 to less than 10%.^6^, ^21^

In 2015, Vandergriff et al. published a study to validate the Pimpinelli et al. algorithm. The authors found 87.5% of sensitivity and 60% of specificity for the diagnosis and concluded that the algorithm is a statistically valid method.^22^

Besides the known improvement in the diagnosis of early stage MF, investigation of TCR gene rearrangement is complex, unavailable in many laboratories and presents differences between detection rates depending on techniques employed, number of samples and MF's phase (patch, plaque or tumor).^23^, ^24^, ^25^ Even without performing these molecular complementary diagnostic test, we found an increase of the percentage of MF diagnosis from 64.2%, when considered only histopathological findings, to 91% when applying the available algorithm criteria. We point out that the algorithm performace could been even better since 6/67 did not achieved enough points, which they could have if TCR rearrangement clonallity test was available and, of course, if the test had been positive.

## Conclusion

Although clinicopathological correlation remains the “gold standard”, application of clinical, histopathological and immunohistochemmicals criteria from the Pimpinelli et al. algorithm was useful in the diagnosis of early MF and can contribute to an improvement of the patient's outcome by offering earlier and specific treatment. Our results motivated the adoption of the algorithm criteria in our practice routine at our Department.

As limitations of the present study we highlight its retrospective character, the reduced sample size and the lack of TCR gene rearrangemnt clonality test.

## Financial support

None declared.

## Authors' contributions

Gustavo Moreira Amorim: Statistic analysis; conception and planning of the study; elaboration and writing of the manuscript; obtaining, analysis, and interpretation of the data; critical review of the literature.

Daniele Carvalho Quintella: Approval of the final version of the manuscript; conception and planning of the study; obtaining, analysis, and interpretation of the data; effective participation in research orientation; critical review of the manuscript.

João Paulo Niemeyer-Corbellini: Conception and planning of the study; intellectual participation in the propaedeutic and/or therapeutic conduct of the studied cases; critical review of the literature.

Luiz Claudio Ferreira: Elaboration and writing of the manuscript; obtaining, analysis, and interpretation of the data; intellectual participation in the propaedeutic and/or therapeutic conduct of the studied cases.

Marcia Ramos e Silva: Approval of the final version of the manuscript; conception and planning of the study; effective participation in research orientation; critical review of the manuscript.

Tullia Cuzzi: Approval of the final version of the manuscript; conception and planning of the study; obtaining, analysis, and interpretation of the data; effective participation in research orientation; critical review of the manuscript.

## Conflicts of interest

None declared.
