# Supplementary material for: Validation of an algorithm based on clinical, histopathological and immunohistochemical data for the diagnosis of early-stage mycosis fungoides
Source: An Bras Dermatol. 2020 Mar 20;95(3):326–31. doi: 10.1016/j.abd.2020.01.002 (PMC8074686; doi:10.1016/j.abd.2020.01.002)
Supplement: Supplementary file 1 [file mmc1.docx]

Supplemental Digital Content to be available online:

**Table 1** Variables studied, summarizing materials and methods.

| **Histopathological variables** | | **Classification** | **Definitions** |
| --- | --- | --- | --- |
| Epidermis | Hyperkeratosis | Qualitative, nominal and dichotomic |  |
|  | Parakeratosis |  |  |
|  | Normal Epidermis | Qualitative and nominal |  |
|  | Thin Epidermis |  |  |
|  | Irregular acanthosis |  |  |
|  | Psoriasiform Acanthosis |  |  |
|  | Vacuolar alteration of the basal layer | Qualitative, nominal and dichotomic |  |
| Lymphoid Infiltrate | Superficial | Qualitative and nominal | According to its predominant distribution pattern |
|  | Perivascular |  |  |
|  | Superficial and Deep Perivascular |  |  |
|  | Lichenoid |  |  |
|  | Confluent and Diffuse |  |  |
| Intraepithelial Lymphocytes | Epidermotropism with or without Pautrier’s microabscesses | Qualitative, nominal and dichotomic | Lymphocytes aligned along the basal layer and upper, in the epidermis, isolated, with halo. Pautrier’s microabscesses: cluster of at least 4 atypical lymphocytes inside the epidermis.[16] |
|  | Folliculotropism without mucinosis |  |  |
|  |  |  | Folliculotropism, equivalent to epidermotropism, but inherent to the epithelium of the hair follicle. |
| Lymphoid Atypia | Convolution of the lymphocyte nucleus in the epidermis and/or dermis  Increase of the size of the lymphocytes nuclei, also in the epidermis and/or dermis | Qualitative, nominal and dichotomic | Lymphoid atypia criteria follow the diagnostic algorithm.[6] Lymphoid nucleus were considered increased when equal or greater than the nuclei of the basal keratinocytes.[17] |
| **Clinical Variables** | | **Classification** | **Definitions** |
| Gender | | Qualitative, nominal and dichotomic | Male or female |
| Age at time of diagnosis | | Quantitative, continuous manner | Years |
| Evolution period | | Quantitative, continuous manner | Months |
| Elementary lesion | | Qualitative, nominal and dichotomic | Macules or plaques, According to its predominant |
| Number of lesions | | Qualitative, nominal and dichotomic | Single or multiple lesions |
| Variation of size and form of the lesions | | Qualitative, nominal and dichotomic |  |
| Topography of the lesions | | Qualitative, nominal and dichotomic | Predominance between non-photoexposed and photoexposed areas |
| Poikiloderma | | Qualitative, nominal and dichotomic | Identification of dyschromia with mottled aspect, telangiectasia and epidermal atrophy.[6] |
| TNMB staging | | Qualitative, nominal and dichotomic | IA or IB.[9] |
| Disease follow-up period | | Quantitative, continuous manner | Years |
| **Immunohistochemical variables** | | **Classification** | **Definitions** |
| Positivity of CD2 | | Discrete quantitative analysis | Markers were measured in terms of percentage for total lesional infiltrate. The loss of <50% for CD2+, CD3+ and/or CD5+ T-cells and <10% for CD7+ T-cells  were considered. |
| Positivity of CD3 | |  |  |
| Positivity of CD5 | |  |  |
| Positivity of CD7 | |  |  |
| Dermoepidermal disagreement | | Qualitative, nominal and dichotomic | Positivity difference between epidermal and dermal lymphocytes.[6] |

**Table 2** Results of the immunohistochemical analysis as part of the aplication of the diagnostic algorhythm of Pimpinelli et al.

| **Case** | **CD3**  **Epi/Derm** | **%** | **CD2**  **Epi/Derm** | **%** | **CD5**  **Epi/Derm** | **%** | **CD7**  **Epi/Derm** | **%** | **Pt.** |
| --- | --- | --- | --- | --- | --- | --- | --- | --- | --- |
| 1 | Pos/Pos | 80 | Neg/Pos | 10 | Neg/Pos | 20 | ‒ | 0 | 1 |
| 2 | Pos/Pos | 90 | Neg/Pos | 10 | Neg/Pos | 70 | ‒ | 0 | 1 |
| 3 | Pos/Pos | 80 | Neg/Pos | 10 | Pos/Pos | 20 | ‒ | 0 | 1 |
| 4 | Pos/Pos | 95 | Neg/Pos | 10 | Pos/Pos | 30 | ‒ | 0 | 1 |
| 5 | Neg/Pos | 90 | Neg/Pos | 10 | Neg/Pos | 10 | ‒ | 0 | 1 |
| 6 | Neg/Pos | 90 | Neg/Pos | 5 | Neg/Pos | 30 | ‒ | 0 | 1 |
| 7 | Pos/Pos | 95 | ‒ | 0 | Pos/Pos | 80 | Pos/Pos | 70 | 1 |
| 8 | Neg/Pos | 40 | ‒ | 0 | Neg/Pos | 10 | ‒ | 0 | 1 |
| 9 | Neg/Pos | 90 | Neg/Pos | 50 | ‒ | 0 | ‒ | 0 | 1 |
| 10 | Neg/Pos | 90 | Neg/Pos | 50 | ‒ | 0 | ‒ | 0 | 1 |
| 11 | Neg/Pos | 30 | Neg/Pos | 5 | Neg/Pos | 10 | Neg/Pos | 30 | 1 |
| 12 | Neg/Pos | 80 | ‒ | 0 | ‒ | 0 | ‒ | 0 | 1 |
| 13 | Pos/Pos | 90 | Neg/Pos | 20 | Pos/Pos | 50 | ‒ | 60 | 1 |
| 14 | Neg/Pos | 90 | ‒ | 0 | Neg/Pos | 20 | Neg/Pos | 40 | 1 |
| 15 | Pos/Pos | 90 | ‒ | 0 | Neg/Pos | 20 | ‒ | 0 | 1 |
| 16 | Pos/Pos | 90 | ‒ | 0 | Neg/Pos | 20 | ‒ | 0 | 1 |
| 17 | Pos/Pos | 80 | Neg/Pos | 10 | Neg/Pos | 5 | ‒ | 0 | 1 |
| 18 | Pos/Pos | 90 | ‒ | 0 | Neg/Pos | 90 | Neg/Pos | 20 | 1 |
| 19 | Pos/Pos | 95 | ‒ | 0 | Neg/Pos | 10 | Pos/Pos | 90 | 1 |
| 20 | Pos/Pos | 95 | ‒ | 0 | Neg/Pos | 80 | Neg/Pos | 50 | 1 |
| 21 | Neg/Pos | 10 | ‒ | 0 | ‒ | 0 | Neg/Pos | 10 | 0 |
| 22 | Neg/Pos | 90 | ‒ | 0 | Neg/Pos | 40 | Neg/Pos | 90 | 1 |
| 23 | Neg/Pos | 50 | ‒ | 0 | Neg/Pos | 5 | Neg/Pos | 20 | 1 |

Epi., Epidermis; Derm., Dermis; Neg., Negative; Pos., Positive; Pt., Points, according to the algorithm analysis.

**Table 3** Immunohistochemical criteria, according to the proposed algorithm.

| **< 50% positive T CD3 cells** | 13.0% | 03/23 |
| --- | --- | --- |
| **< 50% positive T CD2 cells** | 91.3% | 21/23 |
| **< 50% positive T CD5 cells** | 78.2% | 18/23 |
| **< 10% positive T CD7 cells** | 56.5% | 13/23 |
| **Dermoepidermal disagreement** | 52.1% | 12/23 |

An overlap of criteria may occur, however, as proposed in the algorithm; the maximum score within the immunohistochemical criteria is 1 (one).
